# Serum Uric Acid, Hypertriglyceridemia, and Carotid Plaques: A Sub-Analysis of the URic Acid Right for Heart Health (URRAH) Study

**DOI:** 10.3390/metabo14060323

**Published:** 2024-06-07

**Authors:** Claudia Agabiti Rosei, Anna Paini, Giacomo Buso, Alessandro Maloberti, Cristina Giannattasio, Massimo Salvetti, Edoardo Casiglia, Valerie Tikhonoff, Fabio Angeli, Carlo Maria Barbagallo, Michele Bombelli, Federica Cappelli, Rosario Cianci, Michele Ciccarelli, Arrigo Francesco Giuseppe Cicero, Massimo Cirillo, Pietro Cirillo, Raffaella Dell’Oro, Lanfranco D’Elia, Giovambattista Desideri, Claudio Ferri, Ferruccio Galletti, Loreto Gesualdo, Guido Grassi, Guido Iaccarino, Luciano Lippa, Francesca Mallamaci, Stefano Masi, Maria Masulli, Alberto Mazza, Alessandro Mengozzi, Pietro Nazzaro, Paolo Palatini, Gianfranco Parati, Roberto Pontremoli, Fosca Quarti-Trevano, Marcello Rattazzi, Gianpaolo Reboldi, Giulia Rivasi, Elisa Russo, Giuliano Tocci, Andrea Ungar, Paolo Verdecchia, Francesca Viazzi, Massimo Volpe, Agostino Virdis, Maria Lorenza Muiesan, Claudio Borghi

**Affiliations:** 1 Department of Clinical and Experimental Sciences, University of Brescia, and ASST Spedali Civili Brescia, Piazzale Spedali Civili 1, 25123 Brescia, Italy; claudia.agabitirosei@unibs.it (C.A.R.); anna.paini@asst-spedalicivili.it (A.P.); giacomo.buso@unil.ch (G.B.); massimo.salvetti@unibs.it (M.S.); 2 Cardiology IV, “A.De Gasperi’s” Department, Niguarda Ca’ Granda Hospital, 20162 Milan, Italy; alessandro.maloberti@ospedaleniguarda.it (A.M.); cristina.giannattasio@unimib.it (C.G.); 3 School of Medicine and Surgery, Milano-Bicocca University, 20126 Milan, Italy; 4 Studium Patavinum, Department of Medicine, University of Padua, 35100 Padua, Italy; edoardo.casiglia@unipd.it (E.C.); palatini@unipd.it (P.P.); 5 Department of Medicine, University of Padua, 35100 Padua, Italy; valerie.tikhonoff@unipd.it; 6 Department of Medicine and Surgery, University of Insubria, 21100 Varese, Italy; fabio.angeli@uninsubria.it; 7 Department of Medicine and Cardiopulmonary Rehabilitation, Maugeri Care and Research Institutes, IRCCS, Istituto di Ricovero e Cura a Carattere Scientifico Tradate, 21100 Varese, Italy; 8 Biomedical Department of Internal Medicine and Specialistics, University of Palermo, 90100 Palermo, Italy; carlo.barbagallo@unipa.it; 9 Clinica Medica, Department of Medicine and Surgery, University of Milano-Bicocca, 20900 Monza, Italy; michele.bombelli@unimib.it (M.B.); raffaella.delloro@unimib.it (R.D.); guido.grassi@unimib.it (G.G.); fosca.quarti@unimib.it (F.Q.-T.); 10 Department of Clinical and Experimental Medicine, University of Pisa, 56126 Pisa, Italy; federica.cappelli@med.unipi.it (F.C.); stefano.masi@unipi.it (S.M.); alessandro.mengozzi@unipi.it (A.M.); agostino.virdis@unipi.it (A.V.); 11 Department of Translational and Precision Medicine, Sapienza University of Rome, 00185 Rome, Italy; rosario.cianci@uniroma1.it; 12 Department of Advanced Biomedical Sciences, “Federico II” University of Naples, 80133 Naples, Italy; mciccarelli@unisa.it (M.C.); guiaccar@unina.it (G.I.); 13 Department Hypertension and Cardiovascular Disease Research Center, Medical and Surgical Sciences Department, Alma Mater Studiorum University of Bologna, 40126 Bologna, Italy; arrigo.cicero@unibo.it (A.F.G.C.); claudio.borghi@unibo.it (C.B.); 14 Heart-Chest-Vascular Department, IRCCS AOU of Bologna, 40126 Bologna, Italy; 15 Department of Public Health, “Federico II” University of Naples, 80133 Naples, Italy; mcirillo@unisa.it; 16 Nephrology, Dialysis and Transplantation Unit, Department of Emergency and Organ Transplantation, “Aldo Moro” University of Bari, 70122 Bari, Italy; pietro.cirillo@policlinico.ba.it (P.C.); loreto.gesualdo@uniba.it (L.G.); 17 Department of Clinical Medicine and Surgery, “Federico II” University of Naples, 80133 Naples, Italy; lanfranco.delia@unina.it (L.D.); galletti@unina.it (F.G.); magia77@inwind.it (M.M.); 18 Department of Clinical, Internal, Anesthesiologic and Cardiovascular Sciences Sapienza, University of Rome, 00161 Rome, Italy; giovambattista.desideri@cc.univaq.it; 19 Department of Life, Health and Environmental Sciences, University of L’Aquila, 67100 L’Aquila, Italy; claudio.ferri@univaq.it; 20 Italian Society of General Medicine (SIMG), 67051 Avezzano, Italy; lippa.luciano@simg.it; 21 CNR-IFC, Clinical Epidemiology of Renal Diseases and Hypertension, Reggio Cal Unit, 89124 Reggio Calabria, Italy; francesca.mallamaci@libero.it; 22 Department of Internal Medicine, Santa Maria Della Misericordia General Hospital, AULSS 5 Polesana, 45100 Rovigo, Italy; alberto.mazza@aulss5.veneto.it; 23 Center for Translational and Experimental Cardiology (CTEC), Department of Cardiology, University Hospital Zurich, University of Zurich, 8952 Schlieren, Switzerland; 24 Sant’Anna School of Advanced Studies, Research University, 56127 Pisa, Italy; 25 Department of Precision and Regenerative Medicine and Jonic Area (DiMePRe-J), Neurosciences and Sense Organs, University of Bari Medical School, 70122 Bari, Italy; pietro.nazzaro@uniba.it; 26 Istituto di Ricovero e Cura a Carattere Scientifico (IRCCS), Istituto Auxologico Italiano, Department of Cardiology, Institute San Luca Hospital, Piazzale Brescia, 20149 Milan, Italy; gianfranco.parati@unimib.it; 27 Department of Medicine and Surgery, University of Milano-Bicocca, Piazza dell’Ateneo Nuovo, 20126 Milan, Italy; 28 Department of Internal Medicine, University of Genoa, IRCSS Ospedale Policlinico San Martino, 16132 Genova, Italy; roberto.pontremoli@unige.it (R.P.); elisa.russo@unige.it (E.R.); francesca.viazzi@unige.it (F.V.); 29 Department of Medicine—DIMED, University of Padova, Medicina Interna 1° Ca’ Foncello University Hospital, 31100 Treviso, Italy; marcello.rattazzi@unipd.it; 30 Department of Medical and Surgical Science, University of Perugia, 06100 Perugia, Italy; paolo.reboldi@unipg.it; 31 Department of Geriatric and Intensive Care Medicine, Careggi Hospital, University of Florence, 50121 Florence, Italy; giulia.rivasi@unifi.it (G.R.); andrea.ungar@unifi.it (A.U.); 32 Department of Clinical and Molecular Medicine, University of Rome Sapienza, 00185 Rome, Italy; giuliano.tocci@uniroma1.it (G.T.); massimo.volpe@uniroma1.it (M.V.); 33 Hospital S. Maria della Misericordia, 06100 Perugia, Italy; auci.perugia@tin.it; 34 IRCCS San.Raffaele, Via della Pisana, 00163, Rome, Italy

**Keywords:** serum uric acid, triglycerides, cardiovascular risk, carotid plaques, HDL cholesterol, LDL cholesterol

## Abstract

High levels of serum uric acid (SUA) and triglycerides (TG) might promote high-cardiovascular-risk phenotypes, including subclinical atherosclerosis. An interaction between plaques xanthine oxidase (XO) expression, SUA, and HDL-C has been recently postulated. Subjects from the URic acid Right for heArt Health (URRAH) study with carotid ultrasound and without previous cardiovascular diseases (CVD) (n = 6209), followed over 20 years, were included in the analysis. Hypertriglyceridemia (hTG) was defined as TG ≥ 150 mg/dL. Higher levels of SUA (hSUA) were defined as ≥5.6 mg/dL in men and 5.1 mg/dL in women. A carotid plaque was identified in 1742 subjects (28%). SUA and TG predicted carotid plaque (HR 1.09 [1.04–1.27], *p* < 0.001 and HR 1.25 [1.09–1.45], *p* < 0.001) in the whole population, independently of age, sex, diabetes, systolic blood pressure, HDL and LDL cholesterol and treatment. Four different groups were identified (normal SUA and TG, hSUA and normal TG, normal SUA and hTG, hSUA and hTG). The prevalence of plaque was progressively greater in subjects with normal SUA and TG (23%), hSUA and normal TG (31%), normal SUA and hTG (34%), and hSUA and hTG (38%) (Chi-square, 0.0001). Logistic regression analysis showed that hSUA and normal TG [HR 1.159 (1.002 to 1.341); *p* = 0.001], normal SUA and hTG [HR 1.305 (1.057 to 1.611); *p* = 0.001], and the combination of hUA and hTG [HR 1.539 (1.274 to 1.859); *p* = 0.001] were associated with a higher risk of plaque. Our findings demonstrate that SUA is independently associated with the presence of carotid plaque and suggest that the combination of hyperuricemia and hypertriglyceridemia is a stronger determinant of carotid plaque than hSUA or hTG taken as single risk factors. The association between SUA and CVD events may be explained in part by a direct association of UA with carotid plaques.

## 1. Introduction

Serum uric acid (SUA) and triglycerides (TG) are strictly related and both involved in the development of cardiovascular damage [1,2]. Both SUA and TG, however, have not been properly included in cardiovascular risk stratification, because interventional trials have shown limited effects resulting from their changes, mainly when compared with LDL-cholesterol. In addition, the role of both UA and TG in favoring CV events may simply reflect the presence of an impairment in control of a metabolic disease [3], although some studies have suggested a possible interplay between the elevated SUA and TG levels [4] both contributing to the activation of the NLRP3-inflammasome [5,6]. Very recently, it was observed that SUA and TG have an independent effect on mortality risk prediction within the framework of the the URic acid Right for heArt Health (URRAH) study; the risk prediction of SUA cut-offs was confirmed even after stratification for TG levels, with the specific cut-offs of SUA being consistent in normotriglyceridemic conditions, at the early stages of the cardiometabolic spectrum [7]. Elevated SUA [8,9,10,11,12,13,14,15] and TG levels [16,17,18,19] have been increasingly investigated as potential novel predictors and risk factors for all-cause and cardiovascular mortality. Despite the established role of elevated levels of TG in CV events [16,17,18], their effect on the development and stability of carotid atherosclerosis has been controversial [20,21,22,23]. In patients with coronary artery disease, higher TG levels were associated with a higher prevalence of thin-cap fibroatheromas in culprit coronary lesions [24]. Furthermore, in individuals with low-to-moderate cardiovascular risk, hypertriglyceridemia has been found to be associated with subclinical atherosclerosis and vascular inflammation, even in participants with normal LDL-C levels [25]. Recent data suggest that SUA levels may play a role in the development and progression of carotid atherosclerosis [26]. A higher expression of uric acid in carotid plaque specimens from symptomatic (including stroke, transient ischemic attack, amaurosis fugax) versus asymptomatic patients was demonstrated. Uric acid expression in carotid plaques was positively correlated with SUA levels and was associated with inflammatory markers expressed in carotid plaques [27]. In symptomatic patients, plaques’ overexpression of xanthine-oxydase (XO) in macrophages was associated with increased SUA and low high-density lipoprotein cholesterol (HDL) levels, suggesting a potential role in carotid plaque destabilization [28]. The role of SUA in influencing the degree of plaque instability was also confirmed by the detection of ultrasound features of vulnerable plaque in elderly patients (reduced atherosclerotic plaque echogenicity) alongside higher SUA levels [29]. The Working Group on Uric Acid and Cardiovascular Risk of the Italian Society of Hypertension has conceived and designed an ad hoc protocol aimed at investigating the association between the levels of lipid parameters and SUA and the presence of a plaque in a subset of the URic acid Right for heArt Health (URRAH) database with available ultrasound evaluations of carotid arteries and absence of previous cardiovascular diseases (CVDs).

## 2. Materials and Methods

### 2.1. Protocol Design

The URRAH project is a multicenter retrospective observational cohort study collecting data obtained from subjects aged 18 to 95 years who were consecutively recruited within the epidemiological network of the Italian Society of Hypertension and involving almost all the Italian regions. Caucasian patients attending hypertension clinics and those enrolled in prospective observational cohort studies were included and followed for a mean period of 10.7 ± 5.4 years (median 11.3 years) up to 31 July 2017. The study protocol has been previously described in detail [13,30]. For all subjects, a standardized set of items were recorded, including demographics, metabolic parameters, smoking habits, systolic and diastolic blood pressure (BP), renal function, history of CV, renal and cerebrovascular disease, concomitant treatments, and outcome. We estimated GFR using the CKD-EPI equation as eGFR = 141 × min (SCr/k,1) a _x max (SCr/k,1) − 1.209 × 0.993 age × 1.159 (if black), where k is 0.7 for female patients and 0.9 for male patients, and a is −0.329 for female patients and −0.411 for male patients; min indicates a minimum of SCr/k or 1, and max indicates a maximum of SCr/k or 1 [31]. We defined renal dysfunction as an eGFR < 60 mL/min, which corresponds to National Kidney Foundation KDOQI (Kidney Disease Outcomes Quality Initiative) stage 3 and 4 kidney disease [32]. Previously identified sex-specific cut-off values (5.6 mg/dL in men and 5.1 mg/dL in women) were used to define the presence of hyperuricemia [13]. Hypertriglyceridemia (hTG) was defined as TG ≥ 150 mg/dL, according to the current ESC guidelines on cardiovascular prevention [33]. LDL cholesterol was calculated according to the Friedewald formula. Obesity was defined as a body mass index (BMI) ≥ 30 Kg/m^2^. Hypertension (HT) was also defined by at least two blood pressure recordings ≥ 140/90 mmHg (systolic/diastolic) [33] or treatment with antihypertensive drugs. Diabetes mellitus (DM) was defined according to ADA criteria [34]. A carotid plaque was defined as a focal abnormal wall thickness (carotid artery intima-media thickness; CIMT) > 1.5 mm or focal CIMT thickening > 50% of the surrounding segment [35].

### 2.2. Ethics

The study data were collected routinely or ad hoc in previously authorized studies. Participants underwent no extra tests or interventions, and there was no impact on participants’ care or outcome. The URRAH study was performed according to the Declaration of Helsinki for Human Research. The processing of the patients’ personal data collected in this study complies with the European Directive on the Privacy of Data. Approval was sought from the Ethical Committee of the Coordinating Center at the Division of Internal Medicine of the University of Bologna (No. 77/2018/Oss/AOUBo). All participants signed informed consent for study participation and the publication of the results.

### 2.3. Study Population

The URRAH project database included a total of 27,078 patients. Only subjects without previous cardiovascular disease, a follow-up period ≥ 1 year (up to 20 years), a measure of SUA and lipid parameter levels at the index date, complete information about CV risk factors, and a carotid ultrasound examination were included in the final analysis (n = 6.209).

### 2.4. Statistical Analysis

All statistical analyses were performed using the SPSS software, version 25 (SPSS Inc., Chicago, IL, USA). The normality of the distributions of the variables was evaluated with the Shapiro–Wilk test; because TG were non-normally distributed, log-transformed values were used in the analyses. Paired comparisons between continuous variables were performed using Student’s *t* test. The Chi-squared test was used to evaluate differences between categorical variables. Four different groups were identified according to SUA and TG levels: (1) nTG/nSUA = normal SUA (<5.6 mg/dL in men and <5.1 mg/dL in women) and TG (<150 mg/dL), (2) nTG/hSUA = hSUA (≥5.6 mg/dL in men and ≥5.1 mg/dL in women) and normal TG (<150 mg/dL), (3) hTG/nSUA = normal SUA SUA (<5.6 mg/dL in men and <5.1 mg/dL in women) and hTG (≥150 mg/dL), and (4) hTG/hSUA = hSUA (hSUA (≥5.6 mg/dL in men and ≥5.1 mg/dL in women) and hTG ((≥150 mg/dL). To evaluate differences among groups’ characteristics, the analysis of variance (ANOVA) was used for continuous data with Bonferroni corrections for multiple comparisons, and the Chi-squared test was utilized to evaluate differences between categorical variables. Logistic regression analysis was conducted with the stepwise method to identify independent variables correlated with the presence of a plaque. Models were adjusted for age, sex, diagnosis of hypertension, obesity, CKD (eGFR < 60 mL/min/m^1.73^), angiotensin-converting enzyme inhibitor treatment, diuretics treatment, and diabetes mellitus.

The results are reported as mean with SD, percentage, or hazard ratio (HR) and 95%CI unless otherwise indicated. Two-sided *p* values below 0.05 were considered statistically significant.

## 3. Results

In Table 1, the demographic and clinical characteristics of the whole study population are reported. Few individuals were treated, and the use of different antihypertensive drugs, statins, and allopurinol are reported in Table 1.

A carotid plaque was identified in 1742 subjects (28%). Individuals with carotid plaque were older, with a higher prevalence of smoking, diabetes mellitus, hypertension, chronic kidney disease, and gout. In addition, BP, BMI, serum creatinine, SUA, glucose, total cholesterol and TG were higher in individuals with carotid plaques, while HDL cholesterol was lower (Table 2).

Upon logistic regression analysis, the levels of TG and SUA predicted carotid plaque (HR 1.09 [1.04–1.27], *p* < 0.001 and HR 1.25 [1.09–1.45], *p* < 0.001, respectively, for TG and SUA) in the whole population, independently of age, sex, diabetes, systolic blood pressure, HDL and LDL-cholesterol, and treatment (Table 3).

Four different groups were identified according to SUA and TG levels: (1) nTG/nSUA = normal SUA and TG, (2) nTG/hSUA = hSUA and normal TG, (3) hTG/nSUA = normal SUA and hTG, and (4) hTG/hSUA = hSUA and hTG (Table 4). Individuals with normal levels of TG and SUA were younger and more frequently women, and they had lower BMI and systolic BP values. Heart rates were lower in patients with normal TG and high SUA as compared with those with hTG and nSUA and both high SUA and TG.

The prevalence of carotid plaque was progressively greater in subjects with normal SUA and TG (23%), hSUA and normal TG (31%), normal SUA and hTG (34%), and hSUA and hTG (38%) (chisquare and *p* for trend 0.0001).

Logistic regression analysis showed that hSUA and normal TG [HR 1.159 (1.002 to 1.341); *p* = 0.001], normal SUA and hTG [HR 1.305 (1.057 to 1.611); *p* = 0.001], and the combination of hUA and hTG [HR 1. 539 (1.274 to 1.859); *p* = 0.001] were associated with a higher risk of plaque after adjusting for sex, age, smoking, diabetes, LDL cholesterol, antihypertensive treatment, obesity, and systolic blood pressure.

## 4. Discussion

The results of this sub-analysis of the URRAH study show that SUA and TG are both associated with the presence of a carotid plaque. Their combination increases the risk of carotid plaque presence, possibly identifying patients at high risk of CV. Our findings could be translated on a practical basis, suggesting that patients with even a modest increase in either UA and/or TG should undergo a carotid ultrasound for carotid plaque evaluation. CV risk is driven by ageing and by impairment of metabolic homeostasis including the development of dyslipidemia, obesity, type 2 diabetes (T2D), and arterial hypertension. SUA is frequently elevated in metabolic diseases [1]. Moreover, a potential interaction between SUA, glucose, and lipid metabolism is supported by pathophysiological and clinical evidence [36,37]. Hyperglycemia and altered levels of circulating lipoproteins have common molecular pathways of damage that are also shared with SUA, although from our results, the effect of both TG and SUA seems to be independent of diabetes diagnosis. Our population has a significant prevalence of obese participants, and obesity may increase the prevalence of dyslipidemia which contributes to atherosclerosis. In this sub-analysis of the URRAH study, obesity was independently associated with the presence of carotid plaques. In our cohort, the prevalence of obesity was higher in women, possibly justifying the findings of a similar prevalence of plaques in men and women; a study including data from 11 prospective cohorts from four European countries [38] showed that men had higher CVD mortality than women across all categories of obesity, although the sex difference in CVD mortality was smaller in obese subjects. A recent review, however, could not confirm the association between obesity and carotid plaques, although waist to hip ratio, a more reliable measure of visceral fat than BMI, was associated with the prevalence of carotid plaques [39]. Elevated SUA and TG levels have been increasingly investigated as potential novel predictors and risk factors for all-cause and cardiovascular mortality. Several epidemiological observations have reported an association between SUA and TG levels [2,40]. This relationship was confirmed in the URRAH study, since we have previously observed a significant interaction between TG and SUA (using the acknowledged SUA cut-offs), and SUA retained its capacity to discriminate between higher and lower all-cause mortality and CV mortality risk independently of TG levels [7]. The relevance of exploring SUA’s predictive potential in conditions of both normo- and hypertriglyceridemia lies in the potential interplay between these two CV risk factors in favoring the development of subclinical damage. The close relationship between SUA and lipid profile abnormalities has been reported in previous studies [41,42,43,44,45,46,47] and confirmed in the URRAH database. The association between SUA and lipids could be explained by several mechanisms [48], including oxidative stress caused during the conversion of hypoxanthine to xanthine by the two final biochemical reactions of SUA production [49]. This reaction may determine mitochondrial dysfunction [50] and citrate release to the cytosol, increasing de novo lipogenesis and triglyceride synthesis. Another potential mechanism is represented by the inhibition of lipoprotein lipase activity induced by SUA in endothelial cells [51] and a subsequent increase in circulating LDL levels. In a Japanese cohort, higher SUA levels were shown to predict the development of high LDL and TG levels during a 5-year follow-up [52]. The lower cut-off values used in this study for defining hyperuricemia could suggest that even relatively modestly increased levels of SUA may determine and/or worsen dyslipidemia and promote proatherogenic lipoprotein alterations and early carotid atherosclerosis. The role of SUA in endothelial activation/dysfunction and promoting proatherogenic inflammation could explain the significant association between SUA and IMT observed in a number of studies, both in otherwise healthy populations [53,54,55,56] and in selected patients with diabetes type 2 [57] or with autoimmune disease [58]. The early exposure and accumulation of SUA during a median follow-up of 9 years in the large Kailuan cohort from China was found to be associated with an increased risk of incident stroke, possibly due, at least in part, to the early and progressive development of vascular damage [59]. A pathophysiological role for uric acid in the pathogenesis of carotid atherosclerosis and possibly in plaque instability was recently suggested by the findings of Nardi et al. [27]. They observed that SUA levels are positively related to the expression of uric acid and of other inflammatory markers in carotid plaques; in addition, patients with a symptomatic cerebrovascular event have a higher concentration of uric acid [27] and higher xantino-oxidase expression in the ipsilateral vulnerable carotid plaque, as well as higher SUA levels compared with asymptomatic patients [28]. The detection of ultrasound features of vulnerable plaque such as low echogenicity in elderly patients with higher levels of SUA further suggests a role of SUA in plaque vulnerability [29]. In addition, vascular smooth muscle cells’ proliferation, favoring the shift towards a synthetic phenotype, vascular re-modeling, and atherosclerosis through proteasome and renin–angiotensin system activation, may be promoted by a slight increase in SUA levels [60]. The association of even a modest elevation of TG with carotid atherosclerotic lesions has been confirmed in a very large meta-analysis with rigorous detection of early vascular alterations [23], and more recent data confirm that elevated TG are a marker of CV risk [16,61], underscoring the importance of early control of TG to an optimal level. No data, however, are currently available on the role of both SUA and TG in carotid plaques. In a large group of Chinese patients at high risk of CV, it was recently found that an elevated SUA level was able to impact the effect of HDL-C on carotid atherosclerosis and potentially affect the relationship between HDL-C and inflammation markers. In the same study, the relationship between HDL-C and inflammatory parameters (WBC count, neutrophil ratio, and CRP level) was weaker in hypeuricemic patients, possibly underscoring the effect of SUA on the inflammatory process.

Our findings should be examined within the context of several potential limitations. This is an observational study with a cross-sectional analysis of the URRAH database, and no cause–effect relationship between SUA, TG, and carotid plaque could be established. Elevated TG and SUA could represent surrogate markers of worse dyslipidemia and renal function, with serum creatinine being higher in patients with higher SUA and TG, although it did not enter in the multivariate analysis. The second limitation is represented by the lack of a detailed information on dietary intake, which is needed in order to evaluate the influence of food/beverages that may affect SUA and TG levels. The use of antihypertensives, statins, and allopurinol treatment, although in a small percentage of subjects, might have influenced the strength of SUA, TG, and carotid atherosclerotic plaque association. Nevertheless, their use has been included in the regression models. The third limitation is the impossibility of distinguishing the different conditions of reduced excretion and overproduction of SUA (due to the lack of data on urinary uric acid) and thus selectively discriminating between the different impacts of these two causes of hyperuricemia. Finally, our results concern Caucasian subjects and cannot be extended to other populations with different demographic and clinical features. The exclusion of patients with previous CV events influenced the mean age of participants. Subjects included in the URRAH database without an ultrasound evaluation of a carotid plaque were younger (mean age 54 + 13 years) and were 46% males, generally having lower BP values (PAS/PAD 141.5 + 22/86 + 13 mmHg), It cannot be excluded that because of young age and lack of associated risk factors, an ultrasound was not performed, thereby limiting the results to middle-aged and older subjects. Finally, the analysis with multiple tests might have resulted in false-positive findings.

## 5. Conclusions

Our findings demonstrate that SUA is independently associated with the presence of carotid plaque and suggest that the combination of hyperuricemia and hypertriglyceridemia is a stronger determinant of carotid plaque than isolated hSUA or hTG. The association between SUA and CVD events may be explained in part because of a direct association with carotid plaque. In the future, further research is needed to establish the causality of the association, addressing the role of possible confounders and enhancing the generalizability of our results.

## Figures and Tables

**Table 1:** Demographic and clinical characteristics and the use of antihypertensive drugs, statins, and allopurinol across the whole study population.

Variables	Whole Database (n = 6209)
Age (years)	61 ± 15
Body mass index (kg/m^2^)	26.8 ± 4.4
Obesity (%)	19.5
Systolic BP (mmHg)	145 ± 22
Diastolic BP (mmHg)	83 ± 12
Heart rate (bpm)	69 ± 12
Serum creatinine (mg/dL)	0.95 ± 0.3
Serum uric acid (mg/dL)	5.2 ± 1.40
Serum glucose (mg/dL)	98 ± 235
Total serum cholesterol (mg/dL)	207 ± 39
Triglycerides (mg/dL)	120 ± 66
Triglycerides (mg/dL) log t	4.67 ± 0.45
HDL serum cholesterol (mg/dL)	54 ± 15
LDL serum cholesterol (mg/dL)	128.8 ± 35
Hemoglobin (g/dl)	14.1 ± 1.3
Hematocrit (%)	42 ± 3.4
Smoking habit (yes, ex%)	20
Diabetes (%)	12
Hypertension (%)	75
Chronic renal disease (%)	10
Gout (%)	2.8
Diuretic use (%)	16
ACE inhibitor use (%)	19
Angiotensin receptor blocker use (%)	18
Calcium channel blocker use (%)	12
Beta-blocker use (%)	13
Statin use (%)	10.5
Allopurinol use (%)	1.5

BP: blood pressure, HDL: high-density lipoprotein; LDL: low-density lipoprotein; ACE: angiotensin-converting enzyme.

**Table 2:** Differences between subjects with and without carotid plaque.

Variables	No Carotid Plaque N = 4467	Carotid Plaque N = 1742	Significance *p* or Chisquare
Age (years)	59 ± 15	65 ± 12	<0.001
Male sex (%)	51	53	0.064
Smoking habit (yes, %)	19	22	0.01
Diabetes (%)	11	14	0.001
Hypertension (%)	71	85	0.001
Chronic renal disease (%)	8.6	14	0.001
Gout (%)	3.3	1.2	0.001
Obesity (%)	19.4	19.4	0.65
Serum creatinine (mg/dL)	0.94 ± 0.3	1.0 ± 0.3	<0.001
Serum uric acid (mg/dL)	5.15 ± 1.4	5.43 ± 1.4	<0.001
Serum fasting glucose (mg/dL)	98 ± 25	101 ± 27	<0.001
Total serum cholesterol (mg/dL)	204 ± 37	213 ± 42	<0.001
Triglycerides (mg/dL) log t	4.64 ± 0.49	4.76 ± 0.46	<0.001
HDL serum cholesterol (mg/dL)	55 ± 15	53 ± 16	<0.001
LDL serum cholesterol (mg/dL)	126.3 ± 32	135.5 ± 40	<0.001

BP: blood pressure, HDL: high-density lipoprotein; LDL: low-density lipoprotein.

**Table 3:** Determinants of carotid plaque presence.

	B	SE	Beta	95% CI	Significance
Sex (male)	0.363	0.082	1.438	1.255; 1.688	<0.001
Age (years)	0.030	0.003	1.030	1.024; 1.037	<0.001
LDL-cholesterol (mg/dL)	0.014	0.001	1.014	1.012; 1.017	<0.001
Obesity (yes)	0.227	0.098	1.255	1.037; 1.520	0.02
High uric acid (yes)	0.164	0.083	1.179	1.001; 1.387	0.048
CKD (yes)	0.435	0.103	1.545	1.263; 1.890	<0.001
High TG (yes)	0.346	0.091	1.414	1.182; 1.691	<0.001
Diuretic treatment (yes)	0.688	0.102	1.989	1.628; 2.431	<0.001
Hypertension (yes)	0.448	0.102	1.566	1.281; 1914	<0.001

LDL: low-density lipoprotein; TG: triglycerides; CKD: chronic kidney disease; CI: confidence interval. Diabetes, smoking, and ACE inhibitor treatment did not enter the model.

**Table 4:** Differences among patients with different levels of SUA and TG upon one-way ANOVA analysis.

Variables	nTG/nSUA n = 3080	nTG/hSUA n = 1737	hTG/nSUA n = 607	hTG/hSUA n = 785	Significance ANOVA *p* between Groups
Age (years)	59 ± 15	62 ± 15 *	62 ± 13 *	61.5 ± 14 *	<0.001
Male sex (%)	46	59 *	50 *	58 *	Chi square < 0.001
Smoking habit (yes, %)	18	21 *	23 *	23 *	Chi square < 0.01
Diabetes (%)	10	13	14 *	15 *	Chi square < 0.001
Hypertension (%)	72	88 *§	72	88 *§	Chi square *p* < 0.001
Chronic renal disease (%)	6	15 *§	7	17 *§	Chi square *p* < 0.001
Gout (%)	1.3	5.4 *§	1	3.2 *§	Chi square *p* < 0.001
Obesity (%)	13	22.3	22.5	34.4 *	Chi square *p* < 0.001
eGFR (mL/min/m^1.73^)	83 ± 18	74.5 ± 19 *	79 ± 18 §#	73 ± 20 *§	<0.001
Serum uric acid SUA (mg/dL)	4.3 ± 0.8	6.4 ± 0.9 *	4.5 ± 0.7 *§#	6.8 ± 1.51 *	<0.001
Serum glucose (mg/dL)	95 ± 24	99 ± 21 *	103 ± 37 *†	103 ± 27 *†	<0.001
Total serum cholesterol (mg/dL)	201 ± 37	202 ± 37	229 ± 39 *†	221 ± 39 *†	<0.001
Triglycerides logt	4.44 ± 0.34	4.57 ± 0.30 *	5.27 ± 0.26 *	5.33 ± 0.30 *	<0.001
HDL serum cholesterol (mg/dL)	58 ± 16	53 ± 14 *	50 ± 15 *†	46 ± 13 *†§	<0.001
LDL serum cholesterol (mg/dL)	126 ± 34	129 ± 33 *	140 ± 38 *†	132 ± 37 *	<0.001

SUA: serum uric acid; TG: triglycerides; BP: blood pressure; HDL: high-density lipoprotein; LDL: low-density lipoprotein. nTG/nSUA = normal SUA (<5.6 and 5.1 mg/dL, respectively, in men and women) and TG (<150 mg/dL). nTG/hSUA = high SUA (≥5.6 and 5.1 mg/dL, respectively, in men and women) and normal TG (<150 mg/dL). hTG/nSUA = normal SUA (<5.6 and 5.1 mg/dL, respectively, in men and women) and high TG (≥150 mg/dL). hTG/hSUA = high SUA (≥5.6 and 5.1 mg/dL, respectively, in men and women) and high TG (≥150 mg/dL). Significance vs. nSUA/nTG * *p* < 0.05, 1. Significance vs. nTG/hSUA † *p* < 0.05. Significance vs. hTG/nSUA **§** *p* < 0.05. Significance vs. hTG/hSUA # *p* < 0.05.

## Data Availability

Data are available upon reasonable request to the investigators. The data are not publicly available due to specific restrictions (as data under Ethical Committee protection).
